# Modulation of adult-born neurons in the inflamed hippocampus

**DOI:** 10.3389/fncel.2013.00145

**Published:** 2013-09-06

**Authors:** Karim Belarbi, Susanna Rosi

**Affiliations:** ^1^Brain and Spinal Injury Center, San Francisco General Hospital, University of California at San FranciscoSan Francisco, CA, USA; ^2^Department of Physical Therapy and Rehabilitation Science, University of California at San FranciscoSan Francisco, CA, USA; ^3^Department of Neurological Surgery, University of California at San FranciscoSan Francisco, CA, USA

**Keywords:** adult neurogenesis, chemokines, cytokines, inflammation, microglia

## Abstract

Throughout life new neurons are continuously added to the hippocampal circuitry involved with spatial learning and memory. These new cells originate from neural precursors in the subgranular zone of the dentate gyrus, migrate into the granule cell layer, and integrate into neural networks encoding spatial and contextual information. This process can be influenced by several environmental and endogenous factors and is modified in different animal models of neurological disorders. Neuroinflammation, as defined by the presence of activated microglia, is a common key factor to the progression of neurological disorders. Analysis of the literature shows that microglial activation impacts not only the production, but also the migration and the recruitment of new neurons. The impact of microglia on adult-born neurons appears much more multifaceted than ever envisioned before, combining both supportive and detrimental effects that are dependent upon the activation phenotype and the factors being released. The development of strategies aimed to change microglia toward states that promote functional neurogenesis could therefore offer novel therapeutic opportunities against neurological disorders associated with cognitive deficits and neuroinflammation. The present review summarizes the current knowledge on how production, distribution, and recruitment of new neurons into behaviorally relevant neural networks are modified in the inflamed hippocampus.

## INTRODUCTION

In the adult mammalian brain, the subgranular zone of the dentate gyrus (DG) is one of the brain regions where robust neurogenesis continues throughout life (Altman and Das, 1965; Eriksson et al., 1998; Spalding et al., 2013). Adult-born neurons have the capacity to migrate into the granule cell layer, to differentiate into mature granule neurons and to functionally integrate into hippocampal neural networks. This process is highly plastic, influenced by environmental and endogenous factors, and it appears to be altered during neuropathological conditions (Parent et al., 1997; Dash et al., 2001; Ekdahl et al., 2003). In this review, we summarize the current knowledge on the plasticity of adult-born neurons in animal models of brain injury associated with neuroinflammation and we discuss the role of activated microglia and the contribution of specific inflammatory factors.

## FROM NEURAL PROGENITORS TO NEURONAL INTEGRATION INTO HIPPOCAMPAL NETWORKS

Hippocampal adult-born neurons originate from neural precursor cells located in the subgranular zone of the DG and these cells have limited self-renewal capacity (Kempermann et al., 2003). While most of the newly generated cells die shortly after generation (Kempermann et al., 1997; Biebl et al., 2000), some of the progeny gives rise to neuroblasts that migrate into the DG granule cell layer where they mature into fully functional granule neurons (Kempermann et al., 2003; Esposito et al., 2005). The new cells that become synaptically integrated, receive inputs from the entorhinal cortex, and send axonal projections to hilar neurons and CA3 pyramidal cells (Markakis and Gage, 1999; Laplagne et al., 2007; Toni et al., 2008) can be activated by various stimuli, including behavioral experience (Jessberger and Kempermann, 2003; Ramirez-Amaya et al., 2006; Kee et al., 2007; Belarbi et al., 2012a) or high-frequency electrical perforant path stimulation (Bruel-Jungerman et al., 2006; Jungenitz et al., 2013). During their maturation process, new neurons differ substantially from existing granule cells. Electrophysiological data show that they exhibit a decreased overall induction threshold for long-term potentiation and enhanced synaptic plasticity compared to older neurons (Schmidt-Hieber et al., 2004; Ge et al., 2007). In response to spatial exploration, new neurons are also more likely to express plasticity-related immediate-early genes (IEGs) such as *Arc* (activity-regulated cytoskeleton-associated protein) or IEGs encoding transcription factors such as *cfos* (Ramirez-Amaya et al., 2006; Kee et al., 2007). Furthermore, numerous studies ablating or enhancing adult neurogenesis have demonstrated that hippocampal adult-born neurons are required for hippocampus-dependent forms of spatial memory (Clelland et al., 2009; Trouche et al., 2009; Goodman et al., 2010; Nakashiba et al., 2012). Collectively, these data indicate that adult-born neurons are more likely than existing granule neurons to be recruited into hippocampal networks that process spatial and contextual information and exert a critical role in hippocampus-dependent functions.

## THE INFLAMED HIPPOCAMPUS AND THE MULTIFACETED ROLE OF MICROGLIA ACTIVATION

Microglia derive from primitive myeloid progenitors and constitute the resident immune system in the brain (Ginhoux et al., 2010; Kierdorf et al., 2013). In the absence of pathological insult, microglia exist in a ramified morphological phenotype termed “resting microglia.” Through their highly motile ramifications resting microglia continuously scan their territorial domain and communicate with the other surrounding cells by distinct signaling pathways (Davalos et al., 2005; Nimmerjahn et al., 2005; Hanisch and Kettenmann, 2007; Kettenmann et al., 2011). Furthermore, microglia transiently make contact with presynaptic boutons, postsynaptic spines, and the synaptic cleft (Wake et al., 2009; Tremblay et al., 2010) and facilitate synapses elimination and pruning, therefore likely contributing to the stability and organization of neural networks (Wake et al., 2009; Tremblay et al., 2010; Paolicelli et al., 2011). As a consequence of brain pathology, microglia respond to pathogen-associated or damage-associated molecules and acquire a reactive profile usually referred as “activated microglia.” Typical morphological changes associated with microglia activation include thickening of ramifications and of cell bodies followed by acquisition of a rounded amoeboid shape (Kettenmann et al., 2011). This process is accompanied by expression of novel surface antigens and production of mediators that build up and maintain the inflammatory response of the brain parenchyma. This response is often associated with the recruitment of blood-born macrophages from the periphery which migrate into the injured brain parenchyma (Schilling et al., 2005; Schwartz and Shechter, 2010). Monocyte-derived macrophages are distinct in nature from resident microglia (for review, see London et al., 2013).

Activated microglia in the brain can operate as damage associated cells, producing a plethora of molecules that are essential for the elimination of pathogens, toxic factors (such as protein aggregates) and cellular debris (following neuronal death for example). By producing neurotrophic and growth factors that are pivotal for tissue repair and renewal they contribute to resolve infection or injury and to restore normal tissue homeostasis (Neumann et al., 2006; Lalancette-Hebert et al., 2007). On the other hand, through the release of proinflammatory cytokines, proteases, and reactive oxygen species they can induce neurotoxicity (Block et al., 2007; Hanisch and Kettenmann, 2007).

One of the brain regions most densely populated with microglia is the hippocampus (Lawson et al., 1990); microglia activation in this region is a common landmark following stimulation with the bacterial endotoxin lipopolysaccharide (LPS; Rosi et al., 2005; Belarbi et al., 2012a,b), ionizing irradiation (Monje et al., 2002, 2003; Rola et al., 2008; Rosi et al., 2008; Belarbi et al., 2013), traumatic brain injury (Piao et al., 2013), brain ischemia (Liu et al., 2007), and kainic acid-induced or pilocarpine-induced brain seizure (Andersson et al., 1991; Borges et al., 2003; Turrin and Rivest, 2004; Vezzani et al., 2008). Microglia activation is also present in various models of neurodegenerative diseases associated with abnormal protein aggregation such as in genetically modified mouse models mimicking Alzheimer’s disease amyloid pathology (APP23: Stalder et al., 1999; Bornemann et al., 2001; PS/APP: Matsuoka et al., 2001; PS1 + APP: Gordon et al., 2002; Tg2576: Frautschy et al., 1998; Benzing et al., 1999; Sasaki et al., 2002) or tau pathology (P301S tau: Bellucci et al., 2004; Yoshiyama et al., 2007; TgTauP301L: Sasaki et al., 2008; Thy-Tau22: Belarbi et al., 2011). Normal aging is also characterized by chronic low-level of inflammation and increased microglia reactivity (Jurgens and Johnson, 2012).

**FIGURE 1:**
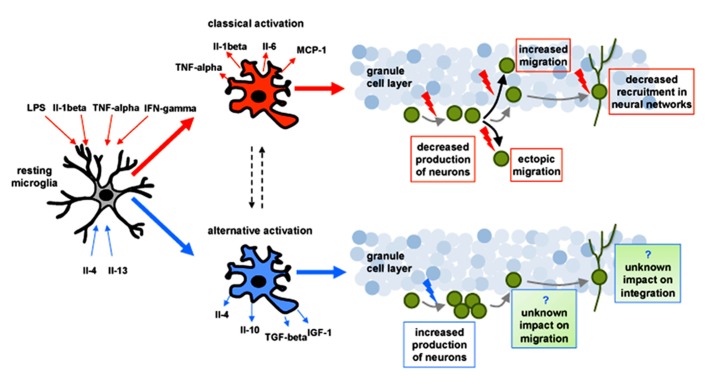
**Schematic drawing representing the impact of classical (depicted in red) and alternative (depicted in blue) activation of microglia on adult-born neurons (depicted in green) in the hippocampus.** In response to changes in the microenvironment microglia can undergo to a potentially neurotoxic “classical activation” (characterized by the release of proinflammatory factors) or a potentially neuroprotective “alternative activation” (characterized by the release of anti-inflamatory cytokines). Polarization toward classical activation can be induced experimentally by exposure to pro-inflammatory cytokines such as (IFN)-gamma, tumor necrosis factor (TNF)-alpha and interleukin (Il)-1beta, as well as bacterial-derived LPS. Classically activated microglia has been shown to: (1) decrease the production of neurons, (2) alter their migration pattern, and (3) reduce their recruitment into neuronal networks (red arrows). Alternative activation of microglia can be induced experimentally by anti-inflammatory cytokines such as Il-4 and Il-13 and can increase the production of neurons (blue arrow). The impact of alternative activation of microglia on the migration and the integration of new neurons remains unknown.

Both macrophages (Porta et al., 2009) and microglia (Michelucci et al., 2009) can undergo different forms of polarized activation leading to a potentially neurotoxic “classic or M1 activation” (characterized by a release of pro-inflammatory factors) or a potentially neuroprotective “alternative or M2 activation” (characterized by anti-inflammatory cytokines). M1 activation is characterized by the release of several proinflammatory and neurotoxic factors including reactive oxygen species, nitric oxide, TNF-alpha, Il-6, Il-1beta, Il-12, and monocyte chemoattractant protein (MCP)-1 (Meda et al., 1996; Kettenmann et al., 2011; Qin et al., 2013). Polarization toward classic activation (M1) can be induced experimentally by exposure to pro-inflammatory cytokines such as interferon (IFN)-gamma, tumor necrosis factor (TNF)-alpha and interleukin (Il)-1beta, as well as bacterial-derived LPS (Lehnardt et al., 2003). Alternative M2 (protective) activation of microglia is characterized by increased expression of the anti-inflammatory cytokines Il-4, Il-10, and transforming growth factor (TGF)-beta, CD200, and growth factors such as insulin growth factor (IGF)-1, nerve growth factor (NGF) or brain-derived neurotrophic factor (BDNF; Butovsky et al., 2005; Yi et al., 2012). Alternative activation can be induced experimentally by anti-inflammatory cytokines such as Il-4 and Il-13 (Butovsky et al., 2006; Colton, 2009). The regulation of this functional polarization after brain injury is still not clear and evidence shows that it should be considered as a dynamic process (Colton, 2009). For example, following ischemia-induced injury in the striatum, microglia initially express the classic activation phenotype, but with time a portion of the cells acquire the alternative activation phenotype (Thored et al., 2009). Therefore, the link between activated microglia and neurogenesis is multifaceted, combining both supportive and detrimental effects dependent upon their phenotype and the factors being released (Butovsky et al., 2006; **Figure 1**).

## PRODUCTION OF NEURONS IN THE INFLAMED HIPPOCAMPUS

Proliferation, differentiation, and survival of neurons in the adult brain has been shown to be modulated in pathological conditions associated with inflammation (Cho and Kim, 2010; Mu and Gage, 2011; Kohman and Rhodes, 2013). Animal models of brain irradiation typically display a significant loss of neural precursor cells that occurs within a few hours (Mizumatsu et al., 2003) and is still present several months after relatively low radiation doses (Tada et al., 2000; Raber et al., 2004a,b; Belarbi et al., 2013). Similarly, neuroinflammation induced by central or systemic administration of LPS significantly reduces basal neurogenesis (Ekdahl et al., 2003; Monje et al., 2003; Fujioka and Akema, 2010), although this is not observed when very low doses of LPS are chronically infused in the ventricular system (Belarbi et al., 2012a). In contrast, increased neuronal production has been reported in animal models of experimental traumatic brain injury (Dash et al., 2001; Kernie et al., 2001; Chirumamilla et al., 2002; Emery et al., 2005; Sun et al., 2007), brain ischemia (Liu et al., 1998; Kee et al., 2001; Yagita et al., 2001; Nakatomi et al., 2002; Choi et al., 2003), and kainic acid-induced or pilocarpine-induced status epilepticus (Parent et al., 1997; Choi et al., 2007). Different animal models of Alzheimer’s disease, provided equivocal data, demonstrating both increased and decreased hippocampal neurogenesis (as reviewed in Mu and Gage, 2011). While differences in many parameters (bromodeoxyuridine administration, cell markers, etc.) could be the cause for these discrepancies, such data provide strong evidence that the modulation of hippocampal adult-born neurons is dependent on the nature of the injury and the time following injury. The initial work investigating the role of activated microglia on neurogenesis found an acute detrimental role for these cells. Classic activation of microglia induced through administration of LPS, either centrally or peripherally, has been shown to block hippocampal neurogenesis (Ekdahl et al., 2003; Monje et al., 2003; Butovsky et al., 2006). In addition, inhibition of microglial activation through administration of minocycline or indomethacin was shown to rescue hippocampal neurogenesis after LPS-induced inflammation (Monje et al., 2003), cranial irradiation (Ekdahl et al., 2003), or focal cerebral ischemia (Hoehn et al., 2005; Liu et al., 2007). In contrast, alternative microglia activation through Il-4 or low level of IFN-gamma could promote neurogenesis (Butovsky et al., 2006). Proinflammatory cytokines released by classically activated microglia can specifically inhibit neural precursor generation, neuronal differentiation, and survival. These include TNF-alpha (Cacci et al., 2005; Heldmann et al., 2005; Iosif et al., 2006), Il-1beta (Goshen et al., 2008; Koo and Duman, 2008; Kuzumaki et al., 2010; Wu et al., 2012), and Il-6 (Vallieres et al., 2002). Conversely, factors released by alternative activation of microglia seem to support the production of neurons as shown for Il-4 (Kiyota et al., 2010), Il-10 (Kiyota et al., 2012), TGF-beta (Battista et al., 2006; Mathieu et al., 2010), and IGF-1 (Choi et al., 2008; Annenkov, 2009). Taken together, these findings suggest that classically activated microglia generally impair neurogenesis whereas alternatively activated microglia promote it, and that these opposite effects are likely dependent upon the specific factors being released (**Figure 1**).

## DISTRIBUTION OF ADULT-BORN NEURONS IN THE INFLAMED HIPPOCAMPUS

In the normal hippocampus neuronal precursors migrate a few micrometers into the granule cell layer where they differentiate into new neurons during the first 2 weeks after production (Kempermann et al., 2003; Seki et al., 2007; Sandoval et al., 2011; Belarbi et al., 2013). Comparative analyses of the distribution of adult-born neurons in different animal models of brain injury suggest that the migration process is altered during pathological conditions. Parent and colleagues first reported ectopic destinations of neural progenitor cells after pilocarpine-induced seizure. Mature neurons were detected not only inside the granule cell layer but also in the molecular layer and inside the hilus of the DG (Parent et al., 1997, 2006). Altered distribution of new neurons within the hippocampus has been also reported in murine models of stroke (Kernie and Parent, 2010), traumatic brain injury (Rosi et al., 2012), cranial-irradiation (Belarbi et al., 2013), and LPS-induced chronic inflammation (Belarbi et al., 2012a). In these models, new neurons were distributed in average a longer distance from the subgranular zone into the granule cell layer. Additional evidence for modified migration of new neurons in the inflamed hippocampus comes from the work of Belmadani et al. (2006) who demonstrated that small cytokine signaling proteins, named chemokines, regulate the migration of neural progenitors to sites of neuroinflammation. In that study neural progenitor cells were grafted into the DG of cultured hippocampal slices and inflammation was achieved by injecting a solution, containing TNF-alpha, IFN-gamma, LPS, glycoprotein 120, or a beta-amyloid-expressing adenovirus, into the area of the fimbria. In control slices, neural progenitors showed little tendency to migrate, while in slices injected with inflammatory stimuli, neural progenitors migrated toward the site of the injection. However, when neural precursors from mice lacking the C–C chemokine receptor type 2 (CCR2 knock-out) were transplanted into slices, they exhibited a greatly reduced migration toward sites of inflammation (Belmadani et al., 2006). CCR2 and its primary ligand MCP-1 are considered to be critical for macrophage trafficking and activation in the brain (Prinz and Priller, 2010). CCR2 has also been shown to be expressed by neural progenitors (Tran et al., 2007). Therefore, these data further support a role for chemokines in the migration of neural progenitor during inflammation. In line with these findings, we recently reported that CCR2 deficiency, through genetic manipulation in mice, was sufficient to prevent the aberrant migration of new neurons observed *in vivo* following irradiation (Belarbi et al., 2013). Similarly, in the pilocarpine-induced status epilepticus rat model, the blockade of the MCP-1/CCR2 interaction with a selective CCR2 antagonist attenuated the ectopic migration of neuronal progenitors into the hilus (Hung et al., 2013). Collectively, these findings indicate that adult-born neurons have the capacity to migrate to the site of damage in response to the chemokine MCP-1/CCR2 signaling pathway. Currently, it is not known whether the change in migration induced by inflammation is beneficial, as, for example, increased migration would allow new neurons to replace dying or lost neurons, or deleterious, as altered migration could reflect the formation of aberrant circuits disrupting hippocampal functions.

## RECRUITMENT OF ADULT-BORN NEURONS INTO BEHAVIORALLY RELEVANT NEURAL NETWORKS IN THE INFLAMED HIPPOCAMPUS

It is widely accepted that induction of effective synaptic plasticity associated with learning and memory requires *de novo* protein synthesis (Miyashita et al., 2008). The IEG *Arc* and its protein are dynamically regulated in response to neuronal activity, and are directly involved in plasticity processes that underlie memory consolidation (Guzowski et al., 2000). The expression of behaviorally induced Arc can be used to study the recruitment of adult-born mature neurons into functional neural networks. Using plasticity-related Arc expression, Ramirez-Amaya and coworkers demonstrated that the proportion of mature new neurons that expressed Arc in response to exploration was significantly higher than the proportion of cells that expressed Arc in the already existing population of granule cells. These data indicate that new neurons are preferentially recruited into hippocampal networks encoding spatial and contextual information (Ramirez-Amaya et al., 2006). In a rat model of LPS-induced chronic neuroinflammation 2-month-old neurons retained the capacity to express behaviorally induced Arc in response to spatial exploration. However, the proportion of new neurons that expressed behaviorally induced Arc was significantly lower than that from sham control animals, indicating that chronic inflammation decreased the recruitment of new neurons into hippocampal networks (Belarbi et al., 2012a). These findings are consistent with the work of Jakubs et al. (2008) that reported an increased inhibitory synaptic drive of new neurons that developed during LPS-induced neuroinflammation. Although adult-born neurons likely contribute to the encoding of recent spatial and contextual information, it is difficult to determine whether decreased excitability of new neurons is beneficial or deleterious to brain function during inflammatory conditions. Indeed, because neuroinflammation was shown to increase the proportion of granule cells expressing behaviorally induced Arc (Rosi et al., 2005), the decrease in new neurons expressing behaviorally induced Arc may be a compensatory mechanism to maintain an optimal level of neuronal activation and ensure the maintenance of pattern separation using a very sparse coding strategy (McNaughton et al., 1996; Rosi, 2011). Arc expression in new neurons as response to behavioral exploration was also reported in mice following exposure to low-dose irradiation combined or not with a subsequent traumatic brain injury in the presence of activated microglia (Rosi et al., 2012). Collectively, these findings show that while new neurons retain the capacity to be recruited into behaviorally relevant neural networks following brain injury, their recruitment is significantly decreased following classical microglia activation.

The chemokine receptor CX3CR1 is present in microglia and circulating monocytes and its unique ligand fractalkine (CX3CL1) is expressed in neurons and peripheral endothelia cells (Bazan et al., 1997; Mizoue et al., 1999). CX3CL1 signaling in the brain promotes microglial survival and controls microglial neurotoxicity through its receptor CX3CR1 under certain neurodegenerative and inflammatory conditions (Garcia et al., 2013). CX3CL1/CX3CR1 signaling is regulated in the inflamed brain, and CX3CR1 is a key regulator of microglia activation contributing to adaptive immune responses (Garcia et al., 2013). Recent evidence demonstrates that in the uninjured brain microglia play a critical role in monitoring and maintaining synapses by directly interacting with synaptic elements (Wake et al., 2009; Tremblay et al., 2010; Paolicelli et al., 2011). Using CX3CR1 knockout mice, Paolicelli et al. (2011) reported a transient reduction in microglial numbers paralleled by a delay in synaptic pruning with consequent excess of dendritic spines and a delayed maturation of excitatory transmission in the developing brain. These results, together with recent data (Rogers et al., 2011; Hoshiko et al., 2012) suggest that CX3CL1/CX3CR1 is an important neuron-microglia signaling pathway necessary for synaptic pruning and maturation (Paolicelli et al., 2011). In light of the role of CX3CL1/CX3CR1 signaling in synaptic maturation together with its involvement in inflammation, it is possible that in the inflamed hippocampus the alteration of this signaling pathway may lead to delayed maturation and/or integration of adult-born neurons. Further studies are needed to better understand how microglia may impact the maturation of adult-born neurons depending of their activation phenotype and the different signaling molecules.

## PERSPECTIVES AND CONCLUDING REMARKS

Available data indicate that the generation, migration, and functional integration of adult-born neurons can be modulated in the inflamed hippocampus, and this modulation appears to differ depending on the activation phenotype of microglia and the specific factors that they release. It is now clear that the range of impact of microglia on adult-born neurons is wider than previously thought, as demonstrated by the anti-neurogenic and pro-neurogenic effects of opposite pro-inflammatory and anti-inflammatory polarized microglia. Previous strategies aimed to maintain functional neurogenesis have mainly focused on decreasing microglia activation. While recent data highlight the potential neuroprotective role of microglia following brain injury, it appears that transforming their phenotype toward alternative activation states could optimize the production, migration, and integration of neurons. Future studies are needed to: (i) characterize the phenotype of microglia activation and the microglia-released factors following brain injury, taking into account the nature of the injury and the timing following the injury; (ii) understand how specific microglia activation states and microglia-released factors impact functional neurogenesis, including migration and functional integration; (iii) identify ways to induce activation of microglia that would support functional neurogenesis in the injured brain. These steps are of critical importance to develop immune-mediated strategies to promote efficient adult-born neurons integration for the maintenance or improvement of hippocampus-dependent cognitive function.

## Conflict of Interest Statement

The authors declare that the research was conducted in the absence of any commercial or financial relationships that could be construed as a potential conflict of interest.
